# Menopause, skin and common dermatosis. Part 3: genital disorders

**DOI:** 10.1111/ced.15400

**Published:** 2022-10-26

**Authors:** Esra Musbahi, Erin Kamp, Mariha Ashraf, Claudia DeGiovanni

**Affiliations:** ^1^ Department of Dermatology University Hospitals Sussex NHS Foundation Trust Brighton UK

## Abstract

Oestrogen plays a vital role in maintaining a normal vulvovaginal epithelium, vaginal lubrication, as well as a healthy microbiome to ensure an acidic pH. The decrease in oestrogen levels in women going through menopause results in both physiological and physical changes of the genitourinary system, and more specifically the vulva. We conducted a literature review on the effects of low oestrogen levels on the physiology and function of the vulva and the vulvovaginal epithelium. ‘Genitourinary syndrome of menopause’ (GSM) is the term used to describe the signs and symptoms of a low oestrogen state. The symptoms and signs of GSM can overlap or coexist with other vulval dermatoses. Expert opinion is needed to diagnose and manage vulval dermatoses in menopause. This article will discuss the signs and symptoms of GSM, as well as the different management options available. Other vulval dermatoses that can be affected by hypo‐oestrogenism are also reviewed.

## Graphical abstract

The low oestrogen levels during menopause lead to physical and physiological changes to the female genitourinary tract. This can lead to the characteristic changes known as genitourinary syndrome of menopause (GSM). The symptoms and signs of GSM can overlap or coexist with other vulval dermatoses. Expert opinion is needed to diagnose and manage vulval dermatoses in menopause.

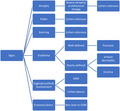

Click here for the corresponding questions to this CME article.

## Introduction

This article reviews the effects of menopause on genital skin, by discussing the features and management of genitourinary syndrome of menopause (GSM), as well as other vulval dermatoses.

## Search strategy

The Cochrane Library, National Institute for Health and Care Excellence (NICE) Evidence database and the Turning Research into Practice database were searched from 2001 to 2021. In total, 116 original research articles were found on menopause in dermatology, 42 of which related to vulval conditions. Individual searches were performed for specific queries related to our paper.

## Oestrogen and the vulva

The embryological origin of the vulva is the primitive urogenital sinus, which is rich in oestrogen receptors, whereas the epithelium of the labia majora is derived from the ectoderm, which has more androgen receptors.^1^ Table 1 illustrates the distribution of hormone receptors in the female genital tract.

**Table 1:** Location and density of hormone receptors in the female genitourinary tract.

Body site	Density of hormone receptors	
	Oestrogen	Androgen
Vagina	++	+
Vulval vestibule	++	+
Labia majora	+	++
Urethra	++	+
Bladder	++	+

Oestrogen is vital in maintaining a normal vulvovaginal epithelium, lubrication and microbiota.^2^, ^3^ Hypo‐oestrogenism leads to pubic hair loss, epithelial atrophy and reduction of vaginal secretions, while loss of collagen and adipose tissue causes architectural changes in the labia.^2^ Reduction of glycogen in the genital epithelium results in more alkaline pH by reducing the number of lactobacilli.^4^, ^5^ The main effects of hypo‐oestrogenism are listed in Table 2.

**Table 2:** Effects of hypo‐oestrogenism on the female genitourinary system.

Reduction in vaginal secretions
Reduction in pubic hair
Reduction in lactobacillus resulting in a more alkaline pH
Epithelial atrophy
Loss of collagen, elastin and adipose tissue leading to architectural change

## Genitourinary syndrome of menopause

GSM is a constellation of symptoms and signs caused by low levels of oestrogen.^1^ These include vaginal dryness, soreness, burning and itching, as well as urinary symptoms. Physical signs include atrophy of the labia and clitoris, and phimosis of the clitoral prepuce. This can affect the physical and mental health, quality of life (QoL), and sexual wellbeing of women going through menopause. GSM is estimated to affect 27–84% of postmenopausal women.^1^ The term ‘genitourinary syndrome of menopause’ was introduced in 2014 to replace older terminology such as ‘vulvovaginal atrophy’ and the negative connotations that come with such descriptions. Diagnosis of GSM is based on symptoms and clinical findings, as well as excluding other alternative causes. Symptom severity does not always correlate with physical findings. It is important to diagnose GSM early, as the symptoms tend to worsen with time in contrast to the vasomotor symptoms of menopause, which tend to resolve eventually for most women.^6^

## Management of genitourinary syndrome of menopause

A holistic and individualized approach is required to manage GSM. The aim of treatment is to relieve the symptoms and improve QoL. It is important to exclude other causes of vulvovaginal symptoms. Treatment can be classified into hormonal or nonhormonal. Nonhormonal topical therapy options are summarized in Table 3.

**Table 3:** Nonhormonal topical management of genitourinary syndrome of menopause.

Type	Examples
Lubricants	
Water‐based	ES WB
	Knect
	Sylk
	Pre‐Seed
	Hanx
	Durex Play water‐based
Silicone‐based	Boots silicone lubricant
	Durex Play silicone based
	Astroglide X
Oil‐based	Natural oils, e.g. olive, coconut
	YES OB
	MegsMenopause
Moisturizers	YES vaginal moisturiser
	Replens MD
	Balance Activ
HA‐based^a^	HYALO GYN
	Revaree
	BioNourish
	GYNTIMA

HA, hyaluronic acid. ^a^Topical and vaginal suppositories/inserts.

## Topical nonhormonal therapy

### Lubricants/moisturizers

Vaginal and vulval lubricants and moisturizers are important first‐line management options to relieve the symptoms of GSM. They help to reduce the friction and discomfort caused by the dryness and atrophy of the genital tissues. A water‐based lubricant is generally preferred with minimal excipients to reduce vulval irritation.^2^

### Hyaluronic acid

Hyaluronic acid (HA) is an important component of the extracellular matrix, and plays a role in the repair of the epidermis and dermis. Menopause causes a reduction of collagen and water retention of the vaginal epithelium. HA is available in various formulations, including gel, vaginal tablets, pessaries and oral tablets.^7^ It can be an option for women who do not want or have contraindications to receiving topical hormonal treatment.^7^

## Hormonal therapy

### Topical

The mainstay of treatment of GSM is topical oestrogen preparation. This has consistently been shown to be the most effective treatment for the symptoms of GSM, especially vulvovaginal atrophy.^1^, ^8^, ^9^ The management of GSM with topical oestrogen is summarized in Table 4.

**Table 4:** Topical oestrogen therapy, management and contraindications.

Type	Comments
Topical oestrogen therapy	The mainstay of topical oestrogen therapy is low‐dose oestrogen
	The two main preparations are oestriol or oestradiol
	Formulations include pessaries, creams and gels, and is determined by symptoms and patient choice
	A Cochrane review found no difference in efficacy for vaginal atrophy between the different formulations of topical oestrogen^21^
	Treatment course is usually once daily for first 2 weeks, then twice weekly for as long as needed
	Response is usually achieved in first 3 months, but might take longer
	Symptoms are likely to return on stopping topical oestrogen therapy
	Topical lubricants and moisturizers should also be used in conjunction with topical oestrogen
Monitoring of therapy	Review response in 3 months and then yearly
	Dose can be increased if no response achieved
	Progesterone is not usually indicated
	Topical treatments are thought to be associated with less risk than systemic HRT
	Unscheduled vaginal bleeding needs to be reviewed
	Routine monitoring of endometrial thickness is not warranted
Women with current or past breast cancer	Women with oestrogen receptor‐positive breast cancer who are taking tamoxifen can be offered low‐dose topical oestrogen therapy if they do not have any other risk factors
	Usually contraindicated in women taking aromatase inhibitors^22^

Topical testosterone (dehydroepiandrosterone) is also used to help with dyspareunia and urinary symptoms of GSM.^10^ Alternative treatments, which can be topical or oral, contain plant‐derived oestrogens (phyto‐oestrogens); these are preferred by some women, but their use is not routinely recommended due to lack of evidence of their efficacy.^1^

### Systemic

#### Systemic hormone replacement therapy

Systemic HRT is an effective treatment for the symptoms associated with menopause including those of GSM.^1^, ^8^ Systemic HRT is not approved by NICE or the US Food and Drug Administrations for the management of GSM alone in the absence of other symptoms of menopause.^1^, ^8^

#### Selective oestrogen receptor modulators

Selective oestrogen receptor modulators are a synthetic nonsteroidal class of drugs, which act on the oestrogen receptor.^1^ is taken orally and is the only agent that has been approved for the treatment of GSM and been shown to be effective compared with placebo.^11^ Further studies are needed to assess safety in patients with breast cancer.

## Device‐based treatment

Lasers and radiofrequency devices have been used to treat the symptoms of GSM.^12^ Resurfacing and/or heat treatment cause remodelling of the genital mucosa by increasing collagen and elastin and promoting neovascularization, so that the genital mucosa is returned to a condition similar to the premenopausal state. Fractional carbon dioxide laser has the most evidence followed by erbium:yttrium–aluminium–garnet, with the least effective being radiofrequency laser.^13^ These treatments can be a useful adjunct for women who cannot use oestrogen therapies, but more randomized controlled studies are needed.

### Physical therapy and lifestyle modifications

Pelvic floor exercises for women who have pelvic floor dysfunction have shown to be effective as measured by improvement in QoL and GSM assessment scores.^14^ Sex therapy for women who have sexual dysfunction as a result of menopause can be of benefit.^1^

## Vulval dermatoses and menopause

**Figure 1:**
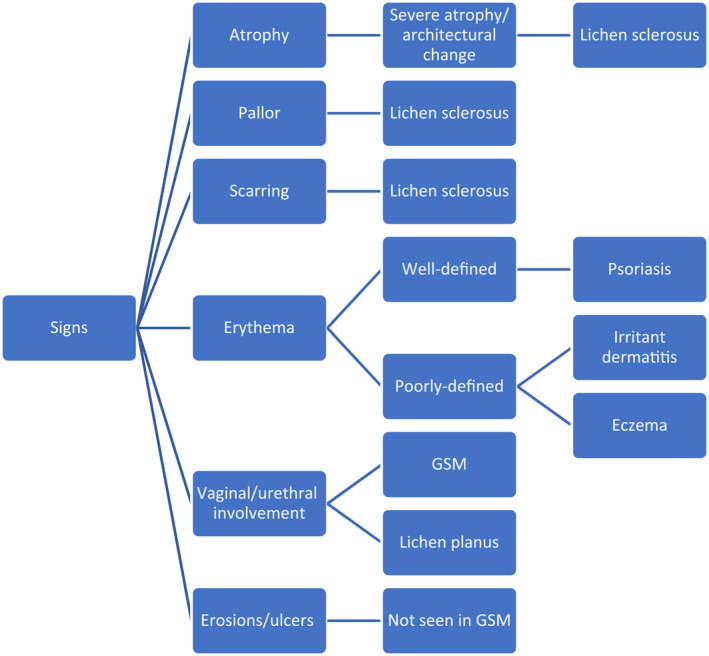
Distinguishing signs of genitourinary syndrome of menopause (GSM) from other vulval dermatoses.

Vulval dermatoses that can present in any age group but can potentially be exacerbated by menopause include lichen sclerosus (LS), lichen planus (LP), irritant dermatitis, and vulvodynia and vulvovaginal candidiasis. Vulval malignancy is also more common with age, and therefore more common in post‐menopausal women.^2^ Figure 1 is a helpful aid to distinguish between the signs of GSM and other vulval dermatoses.

### Vulval lichen sclerosus

Vulval LS is an inflammatory scarring dermatosis that predominantly affects postmenopausal women. The true aetiology is unknown but an autoimmune cause and a genetic predisposition are implicated.^2^ Severe disease results in architectural changes such as fusion of the labia, clitoral tethering and narrowing of the introitus. It can be difficult to differentiate between the changes seen in GSM and LS, and women can have an element of both.^15^ LS is associated with an increased risk of squamous cell carcinoma.^2^ The effect of HRT on the clinical course of LS has not been examined.

### Lichen planus

LP is a mucocutaneous autoimmune condition. It can cause irreversible architectural change and significant morbidity.^16^ The average age of onset of vulval LP correlates with the perimenopause.^13^ It is less common than LS, but it remains an important differential diagnosis as symptoms of both diseases can overlap.

### Vulval dermatitis

The different types of dermatitis that can affect the vulva are atopic dermatitis, irritant contact and allergic contact dermatitis. Urinary incontinence in postmenopausal women can make them susceptible to irritant dermatitis.^3^ Hypo‐oestrogenism leads to lowered water retention ability and an impaired skin barrier function.^17^ This could lead to an increased risk of an irritant dermatitis. Management is education on care of the vulval skin and cessation of the irritant.

### Vulvodynia

Vulvodynia is a chronic pain syndrome in the absence of an identifiable cause.^2^ It can be overlooked in menopausal women. It is usually concomitant with GSM. Although the management options of GSM help with the symptoms of vulvodynia, a holistic approach to management is essential.^18^

### Vulvovaginal candidiasis

The low oestrogen state in menopause means that candidal infections are less likely, and studies have found that vulvovaginal candidiasis is more common in women taking HRT.^19^

### Vulval malignancy

Premalignant and malignant conditions that can affect the vulva are listed in Table 5. The incidence increases with age, and therefore is higher in postmenopausal women.^2^, ^20^

**Table 5:** Premalignant and malignant conditions of the vulva.

Vulval intra‐epithelial neoplasia
Bowen's disease
Squamous cell carcinoma
Extramammary Paget disease
Vulval melanoma

## Conclusion

Menopause and low oestrogen states lead to physical and physiological changes in the genital skin, leading to significant morbidity. It is important to recognize the changes associated with menopause in order to offer appropriate treatment and improve QoL for affected women. More research is needed on the role of hypo‐oestrogenism on vulval conditions and the potential role of HRT.

**Learning points**

- Oestrogen is vital in maintaining a healthy female genitourinary system, and the low levels of oestrogen in menopause results in characteristic changes.
- Decreasing levels of oestrogen in menopause lead to physical and physiological changes in the vulva, which can present as vulvovaginal atrophy.
- GSM is the specific diagnosis given to the effects of the low oestrogen state of menopause on the genitourinary system.
- This review summarizes the effects of menopause on the vulva, including the specific changes associated with GSM, and giving an overview of the management of GMS, along with a discussion of other dermatoses affecting the vulva.
- The mainstay of treatment of GSM is topical oestrogen therapy, which is best used in conjunction with water-based lubricants and moisturizers; systemic HRT is not routinely indicated for the symptoms of GSM alone.
- Dermatoses affecting the vulva include LS, LP and irritant dermatitis; the symptoms of these can overlap with GSM.
- A holistic and multidisciplinary team approach is needed to improve QoL for postmenopausal women.

## Conflict of interest

The authors declare that they have no conflict of interest.

## Funding

None.

## Ethics statement

Ethics approval and informed consent not applicable.

## CPD questions

### Learning objective

To gain up‐to‐date knowledge on the female genitourinary system and disorders that can be associated with menopause.

### Question 1

Which of the following is correct regarding the embryological origins of the female urogenital tract and the healthy vulvovaginal epithelium?

(a) The labia minora is derived from the ectoderm.
(b) The ectoderm has more androgen receptors.
(c) The labia majora originates from the primitive urogenital sinus.
(d) The epithelium of the labia minora is lacking in oestrogen receptors.
(e) An alkaline pH is ideal for the maintenance of a healthy vulvovaginal epithelium and microbiota.

### Question 2

Which of the following statements about genitourinary syndrome of menopause (GSM) is correct?

(a) Atrophy of the vulval tissues is the only physical sign of GSM.
(b) GSM affects a small minority of menopausal women.
(c) Symptom severity usually correlates with physical findings.
(d) The symptoms and signs of GSM can have a significant impact on patient quality of life (QoL).
(e) The symptoms of GSM tend to get better with time.

### Question 3

Which of the following statements about the management of genitourinary syndrome of menopause (GSM) is correct?

(a) Systemic hormone replacement therapy (HRT) is indicated for the management of the vulval symptoms of menopause alone in the absence of other symptoms of menopause such as vasomotor symptoms.
(b) Topical lubricants and moisturizers are not routinely recommended for the relief of symptoms of GSM.
(c) There is no evidence that hyaluronic acid (HA) preparations are useful in the management of the vulval symptoms of GSM.
(d) Ospemifene acts on the progesterone receptors.
(e) There is not enough evidence for laser‐based treatment to routinely recommend their use for the management of GSM.

### Question 3

Topical oestrogen therapy is first‐line management of the genitourinary syndrome of menopause (GSM); which of the following statements is correct?

(a) Topical oestrogen therapy should be continued as long as it is needed.
(b) Regular monitoring of endometrial thickness is advised for women on long‐term topical oestrogen therapy.
(c) Topical oestrogen therapy has rapid onset for the relief of the symptoms of GSM.
(d) Topical oestrogen therapy is safe in women taking aromatase inhibitors for the treatment of breast cancer.
(e) Gel‐based preparations are superior.

### Question 4

Which of the following statements about vulval dermatoses and menopause is correct?

(a) It is easy to differentiate between the clinical findings of vulval lichen sclerosus (LS) and late genitourinary syndrome of menopause (GSM).
(b) Genital candidiasis is more common in menopause.
(c) Low oestrogen levels lead to impaired water retention in the epidermis and impaired skin barrier function.
(d) Vulvodynia usually has a clear cause.
(e) Menopausal women have the same risk as premenopausal women of developing a vulval malignancy.

## Instructions for answering questions

This learning activity is freely available online at http://www.wileyhealthlearning.com/ced

Users are encouraged to

- Read the article in print or online, paying particular attention to the learning points and any author conflict of interest disclosures.
- Reflect on the article.
- Register or login online at http://www.wileyhealthlearning.com/ced and answer the CPD questions.
- Complete the required evaluation component of the activity.

Once the test is passed, you will receive a certificate and the learning activity can be added to your RCP CPD diary as a self‐certified entry.

This activity will be available for CPD credit for 2 years following its publication date. At that time, it will be reviewed and potentially updated and extended for an additional period.

## Data Availability

Not applicable as no datasets were generated or analysed during the current study.
